# Greater radial tuberosity size is associated with distal biceps tendon rupture: a quantitative 3-D CT case–control study

**DOI:** 10.1007/s00167-021-06722-5

**Published:** 2021-09-04

**Authors:** Nick F. J. Hilgersom, Myrthe Nagel, Stein J. Janssen, Izaäk F. Kodde, Bertram The, Denise Eygendaal

**Affiliations:** 1 Department of Orthopaedic Surgery, Amsterdam University Medical Centres, Location AMC, Meibergdreef 9, 1105 AZ Amsterdam, The Netherlands; 2 Department of Orthopaedic Surgery, Amphia Hospital, 4819 EV Breda, The Netherlands; 3 Department of Orthopaedic Surgery, St Antonius Hospital, 3543 AZ Utrecht, The Netherlands

**Keywords:** Elbow, Biceps tendon, Rupture, Radial tuberosity, Size, Morphology, CT, 3D, Distal biceps tendon, Q3DCT, Impingement

## Abstract

**Purpose:**

During pronation, the distal biceps tendon and radial tuberosity internally rotate into the radioulnar space, reducing the linear distance between the radius and ulna by approximately 50%. This leaves a small space for the distal biceps tendon to move in and could possibly cause mechanical impingement or rubbing of the distal biceps tendon. Hypertrophy of the radial tuberosity potentially increases the risk of mechanical impingement of the distal biceps tendon. The purpose of our study was to determine if radial tuberosity size is associated with rupturing of the distal biceps tendon.

**Methods:**

Nine patients with a distal biceps tendon rupture who underwent CT were matched 1:2 to controls without distal biceps pathology. A quantitative 3-dimensional CT technique was used to calculate the following radial tuberosity characteristics: 1) volume in mm^3^, 2) surface area in mm^2^, 3) maximum height in mm and 4) location (distance in mm from the articular surface of the radial head).

**Results:**

Analysis of the 3-dimensional radial tuberosity CT-models showed larger radial tuberosity volume and maximum height in the distal biceps tendon rupture group compared to the control group. Mean radial tuberosity volume in the rupture-group was 705 mm^3^ (SD: 222 mm^3^) compared to 541 mm^3^ (SD: 184 mm^3^) in the control group (*p* = 0.033). Mean radial tuberosity maximum height in the rupture-group was 4.6 mm (SD: 0.9 mm) compared to 3.7 mm (SD: 1.1 mm) in the control group, respectively (*p* = 0.011). There was no statistically significant difference in radial tuberosity surface area (ns) and radial tuberosity location (ns)**.**

**Conclusion:**

Radial tuberosity volume and maximum height were significantly greater in patients with distal biceps tendon ruptures compared to matched controls without distal biceps tendon pathology. This supports the theory that hypertrophy of the radial tuberosity plays a role in developing distal biceps tendon pathology.

**Level of evidence:**

Level III.

## Introduction

Distal biceps tendon ruptures are more common than previously thought with an incidence of approximately 1.2–5.4 per 100,000 persons per year [8, 19]. Risk factors are male gender, smoking, use of steroids, and obesity [8, 19]. However, the exact pathophysiology of distal biceps tendon rupture remains unclear.

During pronation, the distal biceps tendon and radial tuberosity internally rotate towards the ulna, reducing the linear distance between the radius and ulna by approximately 45–48% [3, 11, 18, 22] and leaving little space (< 1 mm) for the biceps tendon to move [14]. This could possibly cause mechanical impingement of the distal biceps tendon. In 1956, Davis and Yassine [4] were the first to suggest but —as far as we know— never proved, that hypertrophic changes of the radial tuberosity decrease the radioulnar space, causing rubbing of the tendon, predisposing it to degenerative changes and finally rupture. Numerous studies have investigated the insertional footprint anatomy of the distal biceps tendon and radial tuberosity morphology to optimize surgical techniques for re-fixation of the distal biceps tendon and functional outcomes [1, 5–7, 15, 16, 20]. However, only Kodde et al. [10] have investigated the possible role of radial tuberosity size in distal biceps tendon rupture. They compared radial tuberosity size between patients with a distal biceps tendon rupture and matched controls without distal biceps tendon pathology using anterior–posterior views of conventional radiographs. Radial tuberosity size was expressed as a ratio based on the maximum diameter of the radius at the radial tuberosity divided by the diameter of the radius just distal of the radial tuberosity. They found no difference in radial tuberosity size. However, their study method leaves room for improvement because the ratio used does not represent absolute radial tuberosity size and the use of 2-dimensional imaging is limited by variation in positioning of the patients’ arm, and thereby incomplete appreciation of the 3-dimensional morphology of the radial tuberosity. Quantitative 3D CT analysis is a more accurate method to assess the morphology, dimensions and volume of bone [9, 17, 24]. Quantitative 3D CT has been used for determining fracture morphology in a wide range of fractures (e.g., glenoid, elbow, and spine fractures) [12, 13, 23, 24], but is also useful for determining lesion size and pattern of osteochondritis dissecans of for example the capitellum [2].

The aim of this study was to assess whether radial tuberosity size plays a role in the pathophysiology of distal biceps tendon ruptures, using quantitative 3D CT analysis. Our null-hypothesis was that there would be no difference in radial tuberosity size (maximum height, surface area, and volume) between patients with distal biceps tendon ruptures and patients without distal biceps tendon ruptures.

## Materials and methods

Adult patients ($\ge$ 18 years of age) with a distal biceps tendon rupture who had a CT scan of the injured elbow before operative re-fixation were included. It is routine practice at our institution to perform a CT scan of the elbow in patients with a delayed presentation of a distal biceps tendon rupture (> 4 weeks of complaints).

Each patient with a rupture (i.e., case) was matched to two control patients without a rupture (i.e., control). Subjects were matched by age within 10-year range and sex. The control group was randomly retrieved from a database of patients who had a CT scan performed of the elbow in the same hospital and based on diagnostic and procedure codes including: elbow trauma (excluding the proximal radius) and degenerative conditions (i.e., osteoarthritis). Indications for control CT scans were: degenerative disease (*n* = 5), trauma (*n* = 12), tumor (*n* = 1).

Patients (cases and controls) were excluded if they had a pre-existing elbow disease believed to affect proximal radius morphology (e.g., congenital disease, trauma).

### Outcome measures and demographics

The medical records were reviewed to identify the baseline characteristics.

**Fig. 1:**
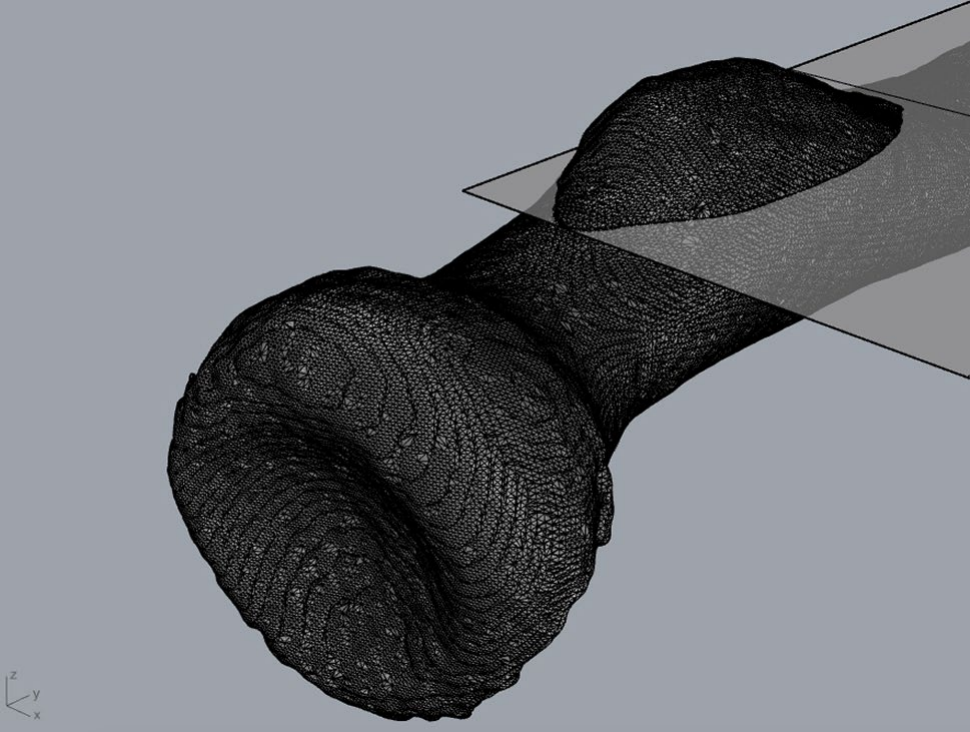
Rhinoceros 3D model of the proximal radius demonstrating the plane created to separate the radial tuberosity from the proximal radial shaft to perform measurements on the radial tuberosity. The *y*-axis determines proximal–distal. The *x*-axis and *z*-axis determine lateral–medial and anterior–posterior, respectively

The outcome measures were: volume, surface area, maximum height, and location of the radial tuberosity. Therefore, 3D models were rendered using OsiriX medical image viewer application (version 10.0.4, Bernex, Switzerland). Cortical bone was automatically identified using a predefined Hounsfield unit value threshold of > 300. The resolution was set highest, decimate-resolution at 0.1, and smooth-iterations at 20 for every case. Subsequently these 3D polygon mesh models were imported in Rhinoceros (McNeel 5.0, Seattle, Washington) for measurements. The position of the 3D models was standardized using the *x*-, *y*-, and *z*-axes in Rhinoceros (Fig. 1).

The shaft of the proximal radius was used to define proximal and distal (*y*-axis), the radial tuberosity was used to define lateral and medial side (*x*-axis), and anterior and posterior (*z*-axis). A plane was created to separate the radial tuberosity from the proximal radius: this was done visually based on the up- and downslope at the beginning and end of the radial tuberosity in the sagittal plane, subsequently the proximal radius was rotated in the axial plane to ascertain that the radial tuberosity lied above the plane (Fig. 1). The researcher (SJJ) who performed the measurements and created the heatmaps was not involved in patient care and blinded for group assignment (case versus control) to avoid bias in measurements. Group assignment was revealed at time of final statistical analysis.

**Fig. 2:**
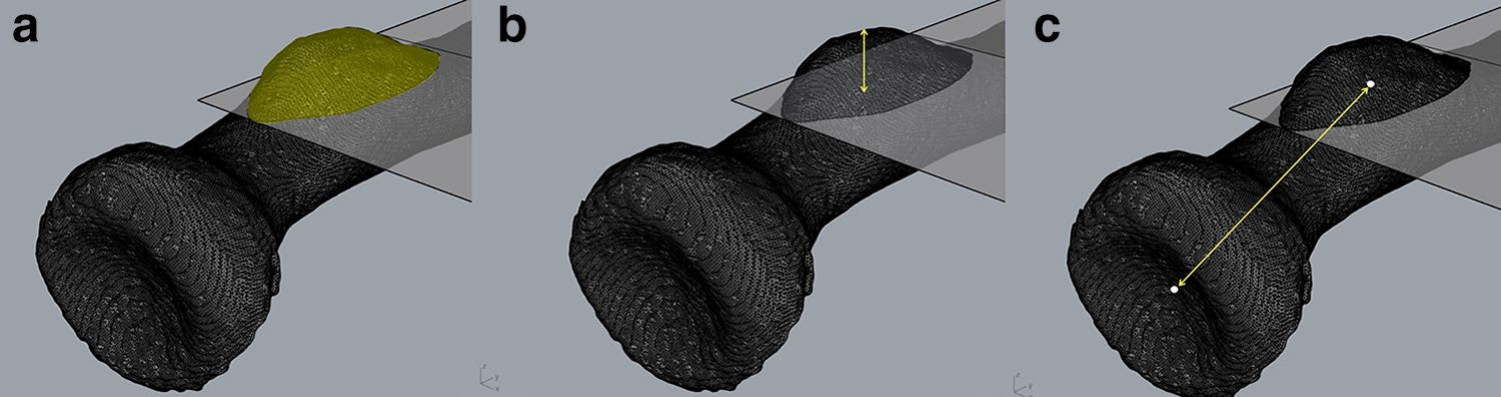
Rhinoceros 3D model of the proximal radius demonstrating the measurements performed on the radial tuberosity. **A** Volume and surface area (marked in yellow), **B** maximum height (greatest distance between the plane separating the radial tuberosity from the proximal radius and top of the radial tuberosity), and **C** location (the distance in mm measured from the volumetric center of the radial tuberosity to the center of the radial head articular surface)

The volume of the radial tuberosity was measured in mm^3^ (mm = millimeter), the surface area in mm^2^ (Fig. 2A), the maximum height in mm (Fig. 2B) and the location as a distance in mm measured from the volumetric center of the radial tuberosity to the center of the radial head articular surface (Fig. 2C). A point grid with *x*, *y*, and *z* coordinates of the radial tuberosity was extracted for further analyses of the radial tuberosity profile. These values were imported per radial tuberosity in Stata 15.0 (StataCorp LP, College Station, TX). Subsequently an averaged radial tuberosity profile heatmap was created for both the cases (distal biceps tendon ruptures) versus controls (no distal biceps tendon rupture) and for each radial tuberosity separately.

Our institutional review board approved this retrospective imaging study (Medical Ethics Committee of the Amphia Hospital; N2018-0131). Informed consent was not required for this retrospective study.

### Statistical analysis

Categorical variables are reported as frequencies and percentages, and continuous variables as mean with standard deviation (SD). To account for paired data, we used mixed-effects linear regression with random effects for the case–control matched groups and fixed effects for the group assignment to calculate *p* values and assess if there was a significant difference in the continuous outcome measures between the cases and the controls. The results of mixed-effects linear regression are interpreted like those derived from a linear regression model.

A two-tailed *p *value below 0.05 was considered statistically significant and Stata was used for all statistical analysis. There were no missing values for any of the variables.

A post hoc power analysis demonstrated that with the included 9:18 matched cases and controls, given the means and standard deviations, and a correlation of 0.1, sufficient power (*β* = 0.85) was achieved to detect a significant difference in volume of the radial tuberosity. In addition, sufficient power (*β* = 0.97) was achieved to detect a significant difference in max radial tuberosity height. Insufficient power (*β* < 0.80) was achieved to detect a significant difference in area (*β* = 0.79) and location (*β* = 0.77) of the radial tuberosity.

## Results

Nine consecutive patients who routinely had a CT scan (0.75–1.00 mm slice thickness, 80 kV, 55–57 mAs) of their elbow performed after a subacute or chronic distal biceps tendon rupture and before operative re-fixation were identified at our institution between October 2015 and March 2018. Five patients had a partial distal biceps tendon rupture, and four patients had a complete distal biceps tendon rupture. No patients had to be excluded based on pre-existing elbow disease believed to affect proximal radius morphology. The cohort consisted of nine cases and 18 sex- and age-matched controls with adequate CT scans for 3D modeling.

The overall mean age was 47 (SD: 10) years for the cases and 46 (SD: 10) years for the controls (Table 1). The age difference was found to be statistically significant (*p* = 0.014). The majority was male: 89% (24/27). There was no statistically significant difference in height, weight or BMI between cases and controls.

**Table 1:** Baseline characteristics of cases (ruptures) and controls (no ruptures)

	Overall (*n* = 27)	Rupture (*n* = 9)	Controls (*n* = 18)	*p* value
	Mean (± SD)	Mean (± SD)	Mean (± SD)	
**Age** (in years)	47 (10)	47 (10)	46 (10)	0.014
**Height** (in cm)*	179 (9.4)	179 (9.1)	179 (10)	ns
**Weight** (in kg)*	82 (16)	86 (17)	79 (16)	ns
BMI*	25 (3.6)	26 (4.1)	24 (3.2)	ns

	*n* (%)	*n* (%)	*n* (%)	*p* value
**Male**	24 (89)	8 (89)	16 (89)	ns
**Right arm affected**	16 (59)	5 (56)	11 (61)	ns

*SD* Standard deviation, *ns* non-significant

*There were 5 missing values for length, weight, and BMI

**Fig. 3:**
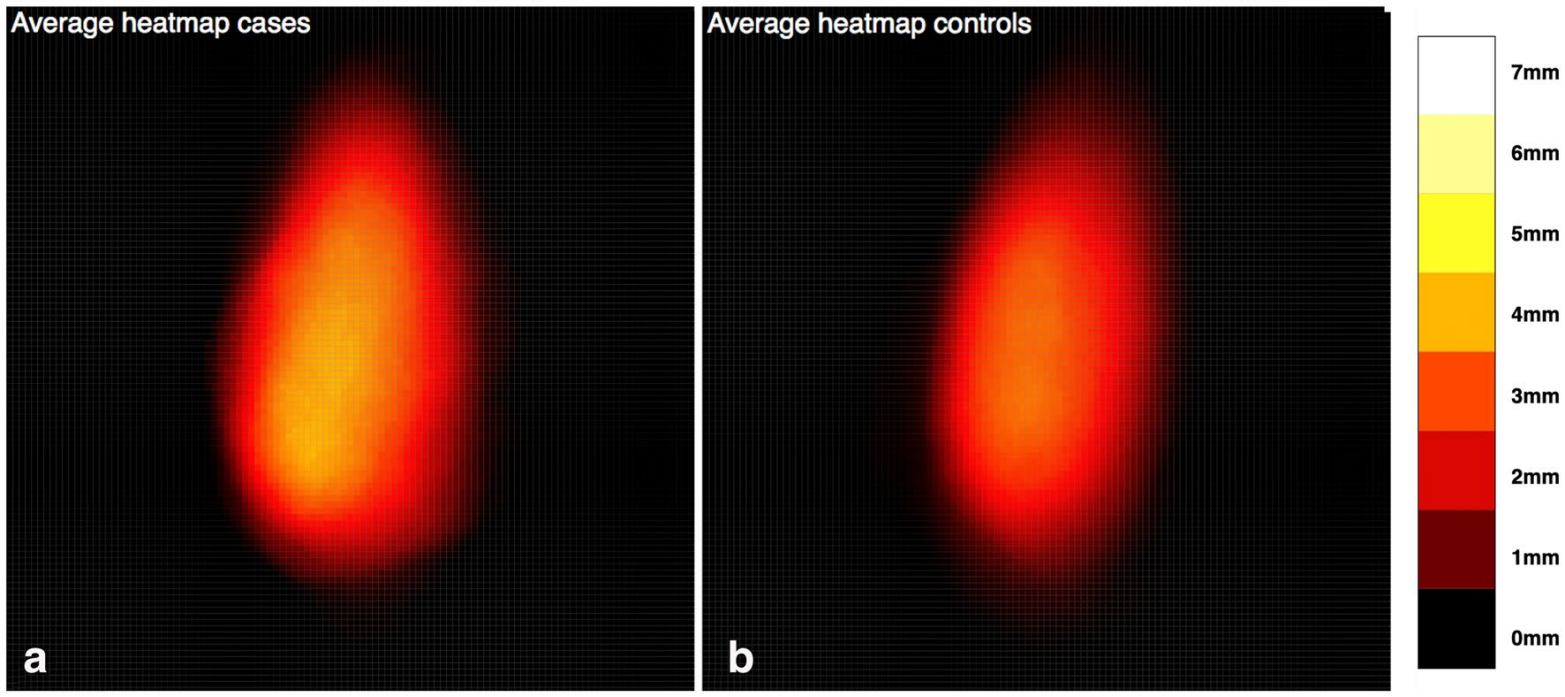
Heatmaps demonstrating the averaged radial tuberosity height profile of cases who had a distal biceps tendon rupture (**A**) versus controls who did not have a distal biceps tendon rupture (**B**). The length of the *x*-axis and *y*-axis are 36.7 mm. The top of the graph is distal, the bottom proximal. All images are right-sided radial tuberosities (left-sided radial tuberosities are mirrored). The color intensity scale indicates the height of the radial tuberosity, 0 mm (black) to 7 mm (white)

Analysis of the 3D radial tuberosity CT-models showed larger radial tuberosity volume and maximum height in the distal biceps tendon rupture group as compared to the control group (Table 2). Mean radial tuberosity volume in the rupture-group was 705 mm^3^ (SD: 222 mm^3^) compared to 541 mm^3^ (SD: 184 mm^3^) in the control group (*p* = 0.033). Mean radial tuberosity maximum height in the rupture group was 4.6 mm (SD: 0.9 mm) compared to 3.7 mm (SD: 1.1 mm) in the control group, respectively (*p* = 0.011) (Fig. 3). There was no statistically significant difference in radial tuberosity surface area (n.s.) and radial tuberosity location (n.s.) with the current sample size.

**Table 2:** Difference in volume, surface area, maximum height and location of radial tuberosity measures between biceps ruptures and controls (no rupture)

	Rupture (n = 9)	Controls (n = 18)	*p* value
	Mean (± SD)	Mean (± SD)	
**Volume** (in mm^3^)	705 (222)	541 (184)	***0.033***
**Area** (in mm^2^)	417 (63)	365 (81)	ns
**Max Height **(in mm)	4.6 (0.9)	3.7 (1.1)	***0.011***
**Location** (in mm)	34 (3.3)	34 (2.4)	ns

*SD* Standard deviation, *ns* non-significant

## Discussion

The presented data, obtained using a quantitative 3D CT technique, show that radial tuberosity volume and maximum height are greater in patients with distal biceps tendon ruptures compared to patients without distal biceps tendon pathology. No significant differences were found for radial tuberosity surface area and location with the current sample size. The greater radial tuberosity volume and maximum height in patients with distal biceps tendon ruptures support the theory that hypertrophic changes of the radial tuberosity may play a role in distal biceps tendon pathology. Therefore, the null-hypothesis that there would be no difference in radial tuberosity volume and maximum height was rejected.

Current results are in contrast to the findings in the study by Kodde et al. [10], who found no significant difference in radial tuberosity size between patients with a distal biceps tendon rupture and matched controls without distal biceps tendon pathology. The difference in results may be explained by two factors: use of a ratio to represent radial tuberosity size, and the used imaging technique. First, Kodde et al. [10] used a ratio to reflect radial tuberosity size that was determined by the maximum diameter of the radius at the radial tuberosity to the diameter of the diaphysis just distal to the radial tuberosity. A drawback of using a ratio is that it is a derivative and not an exact representation of size, therefore less accurate. Second, measurements were performed on conventional AP-views of the elbow, which only provides information in a 2-dimensional plane. Although the method of obtaining the AP-views was standardized, the measured radial tuberosity height would be depending on the angle at which the radial tuberosity was depicted, which can be influenced by patient’s forearm position, anatomic variability in radial tuberosity location between patients and angle of the X-ray beam. Maybe with use of fluoroscopy, it would have been possible to consistently get the plane at which the radial tuberosity is cut at its maximum height; any other angle would show a smaller tuberosity height and lead to underestimation of radial tuberosity size. In this study, both concerns were addressed using 3D CT analysis which provided an accurate 3D-model of the radial tuberosity allowing exact measurements of radial tuberosity size, including height, surface and volume, as well as morphology assessment.

Based on previously published literature, we believe the role of the radial tuberosity in distal biceps tendon pathophysiology can be twofold; causative or reactive. A larger and higher radial tuberosity may directly cause rubbing of the distal biceps tendon during pronation [4], or narrow the radioulnar space causing the distal biceps tendon to become wedged in between the radial tuberosity and lateral ulna [3, 14, 22]. The latter has also been described as a risk factor for re-rupture after distal biceps tendon reconstruction [11]. Both ways of mechanical impingement can cause recurring micro-trauma to the distal biceps tendon, predisposing it to degenerative changes and finally rupture. Reversely, one can also reason from Wolff’s law [25] that the hypertrophic changes of the radial tuberosity are caused by overload of the biceps muscle causing micro-trauma to the distal biceps tendon and radial tuberosity due to traction forces. This may eventually lead to a vicious circle in which micro-trauma causes the radial tuberosity to increase in size, and the hypertrophic radial tuberosity in turn attenuates the distal biceps tendon.

The involvement of radial tuberosity volume and maximum height in distal biceps tendon pathophysiology may have consequences for distal biceps tendon repair technique: i.e., site of tendon reattachment, choice of re-fixation technique and whether or not to reduce radial tuberosity height. One could reason that radial tuberosity height should be reduced during surgery, as such increasing radioulnar space, to prevent mechanical impingement of the reconstructed biceps tendon. However, Schmidt et al. [21] have shown in a cadaveric study that a 25% loss of radial tuberosity height resulted in a significant 27% lower supination moment arm at 60° supination. The absolute difference in maximum height between the rupture group and control group is 0.9 mm (4.6–3.7 mm), this is approximately 20–24% of the radial tuberosity height. Whether this would result in a clinically relevant loss of supination force was not investigated. Therefore, simply reducing the height of the radial tuberosity in all cases is not an option and further investigation is needed.

This study has some limitations. First, this study is limited by a relatively small number of patients which could induce selection bias and limit power. However, CT scanning is performed routinely in patients with chronic distal biceps ruptures at our institution and patients were included in a consecutive fashion reducing the chance of selection bias. Next, quantitative 3D CT analysis is highly accurate limiting the required sample size. To maximize statistical power, cases were matched to controls on a 1:2 basis. A post hoc power analysis demonstrated sufficient power to detect a significant difference in volume and height of the radial tuberosity, and insufficient power to detect a significant difference in area and location of the radial tuberosity. A slightly larger sample size might have resulted in significant differences in radial tuberosity area and location. A significant but small, clinically non-relevant, age difference of 1 year was found between the controls and cases. This was possible as subjects were matched by age within a 10-year range. Second, this is a retrospective study that does not consider all possible confounders. To limit this, matching was performed on the most common available confounders; age and gender. Unfortunately, it was not possible to match for arm dominance, smoking or BMI (height and weight) as these variables were not available for controls in the CT database prior to matching. These factors should be included in future (prospective) studies.

The strength of the current study is the use of a quantitative CT measurement technique that allowed accurate 3D assessment of radial tuberosity characteristics (volume, surface area and position) [9, 17, 24].

This is the first study that shows that radial tuberosity size is associated with the occurrence of distal biceps tendon ruptures, which may have implications for surgical reconstruction (whether or not to reduce radial tuberosity height) and understanding of distal biceps tendon rupture pathophysiology. Whether the role of the radial tuberosity is causative (i.e., mechanical impingement), reactive (i.e., hypertrophy due to recurring micro-traumata) or a combination of both remains to be clarified and warrants further investigation.

It is recommended to assess the radial tuberosity during the surgical procedure, prior to reinsertion, for hypertrophy. Remove any hypertrophic changes, spurs or cortical irregularities if present, but not simply to reduce radial tuberosity height.

## Conclusion

Radial tuberosity volume and maximum height were significantly greater in patients with chronic distal biceps tendon ruptures compared to matched controls without distal biceps tendon pathology. This supports the theory that hypertrophy of the radial tuberosity plays a role in developing distal biceps tendon pathology.
